# The impact of metabolic syndrome on survival outcomes in urothelial carcinoma: a retrospective cohort study

**DOI:** 10.3389/fonc.2026.1858817

**Published:** 2026-06-30

**Authors:** Aspasia Manta, Afroditi Roumpou, Maria Gerogianni, Roubini Zakopoulou, Kimon Tzannis, Areti Mamali, Vasiliki Malamatini, Konstantina Kakogianni, Aristotelis Bamias, Melpomeni Peppa

**Affiliations:** 1 Endocrine Unit, Second Propaedeutic Department of Internal Medicine, Research Institute and Diabetes Center, Attikon University Hospital, School of Medicine, National and Kapodistrian University of Athens, Athens, Greece; 2 Second Propaedeutic Department of Internal Medicine, Research Institute and Diabetes Center, Attikon University Hospital, School of Medicine, National and Kapodistrian University of Athens, Athens, Greece; 3 Third Department of Internal Medicine, Sotiria General Hospital for Chest Diseases School of Medicine, National and Kapodistrian University of Athens, Athens, Greece

**Keywords:** bladder cancer, diabetes mellitus, dyslipidemia, hypertension, metabolic syndrome, obesity, survival, urothelial carcinoma

## Abstract

**Background/objectives:**

Urothelial carcinoma (UC) is a prevalent malignancy of the urinary system, associated with substantial morbidity and mortality. Metabolic syndrome (MetS), comprising obesity, hypertension, dyslipidemia, and hyperglycemia, has been implicated in cancer development, but its prognostic impact in UC remains unclear. This study aimed to explore the association between MetS and survival outcomes in UC patients.

**Methods:**

A single-center retrospective study was conducted, analyzing 112 patients with UC, diagnosed between January 2018 and February 2024. Patients were categorized based on the presence of MetS (≥ 3 criteria) or its individual components. Overall survival (OS) and progression-free survival (PFS) were evaluated using Kaplan–Meier estimates and Cox proportional hazard regression models, adjusting for disease stage and relevant covariates.

**Results:**

The cohort was predominantly male (82.1%), with a median age of 71.9 years (IQR 65.8-79). MetS was present in 49 patients (43.8%). After adjustment for stage, MetS was associated with poorer OS (HR 2.04, p=0.018) and PFS (HR 2.13, p=0.024). In multivariate analysis, MetS independently predicted OS (HR 2.27, p=0.038). Diabetes was the strongest individual predictor of adverse outcomes (adjusted HR for OS 3.33, p=0.001; PFS 3.35, p=0.002). Hypertension also independently predicted worse OS (HR 1.96, p=0.039), whereas obesity and dyslipidemia were not associated with survival.

**Conclusions:**

MetS and its components, particularly diabetes and hypertension, were associated with poorer survival outcomes in patients with UC. Further prospective studies are required to determine whether optimization of metabolic health can improve oncological outcomes.

## 1 Introduction

Urothelial cancer (UC) is one of the most common malignancies worldwide (1). It arises predominantly in the urinary bladder (UBUC), whereas upper tract UC (UTUC) represents 5-10% of cases. The global incidence of UBUC is estimated at 18.2 per 100,000 individuals annually (2), with a male predominance and a median age at diagnosis of approximately 73 years. UTUCs are rarer, affecting about 2 per 100,000 annually (2). Although 70% of newly diagnosed patients present with non-muscle invasive disease, muscle-invasive and metastatic disease are associated with substantially worse outcomes (2). This clinical heterogeneity has prompted efforts to refine disease classification and prognosis, including the definition of oligometastatic disease (3).

Established risk factors for UC include older age, male sex, smoking, occupational exposures, chronic bladder inflammation, and recurrent infections. Dietary habits, particularly high consumption of red and processed meats, and certain genetic conditions also increase the risk, while environmental factors such as aristolochic acid exposure have been linked to UTUC in endemic regions (4). Nonetheless, these factors do not fully explain UC incidence or prognosis, highlighting the need to investigate additional determinants of disease behavior.

Metabolic syndrome (MetS), defined by the presence of at least three components among central obesity, hypertension, dyslipidemia, and diabetes, is recognized as a major contributor to both cardiovascular and cancer pathogenesis and outcomes (5). Epidemiological evidence supports an association of MetS with increased incidence and severity of UC (6). Diabetes, hypertension, hypercholesterolemia, and abdominal obesity have also been linked to UC (7, 8). However, data on the prognostic impact of MetS and its individual components on survival outcomes in UC remain limited.

Given the growing prevalence of MetS and its potential links to UC progression, understanding its impact on patient prognosis is of considerable clinical relevance. In this study, we retrospectively analyzed a cohort of UC patients managed at a large tertiary center to examine the association between MetS, its individual components, and survival outcomes.

## 2 Materials and methods

### 2.1 Study design and population

We conducted a single-center retrospective cohort study including patients with histologically confirmed urothelial carcinoma (UC) managed at the Oncology Department of Attikon University Hospital, Athens, Greece, between January 2018 and February 2024. Eligible participants were ≥18 years old with available data on MetS components and survival outcomes. Patients with non-urothelial histology or incomplete records were excluded.

The primary endpoints of our analysis were overall survival (OS) and progression-free survival (PFS), evaluating the prognostic role of MetS as a syndrome and of its individual components.

The study was approved by the Institutional Review Board at the site (protocol reference number: 448; 14/6/2023), and all patients provided written informed consent to participate based on the principles of the Declaration of Helsinki.

### 2.2 Data collection

Patients’ medical records were reviewed to collect data on demographics, clinical features, disease characteristics, laboratory values, treatment approaches, and outcomes. The stage of disease was determined using the eighth edition of the American Joint Committee on Cancer (AJCC) criteria (9). Patient functional status was evaluated using the Eastern Cooperative Oncology Group Performance Status (ECOG PS) scale (10).

MetS was defined by the presence of three or more of the following factors:

1. Diabetes: Fasting plasma glucose ≥ 100 mg/dL, or ongoing treatment.
2. Dyslipidemia: High-density lipoprotein (HDL) cholesterol <40 mg/dL in men or <50 mg/dL in women; triglycerides ≥150 mg/dl, or ongoing treatment.
3. Hypertension: Blood pressure ≥130/85 mmHg, or ongoing treatment.
4. Obesity: Central obesity is conventionally defined by waist circumference; however, these data were not consistently available in our records. Therefore, body mass index (BMI) ≥ 30 kg/m² was used as a surrogate measure of obesity, consistent with previous retrospective studies in which waist circumference measurements were unavailable (11–13).

Based on these components, patients were grouped into:

1. 0–2 MetS components.
2. ≥3 MetS components (full MetS).

### 2.3 Statistical analysis

Data consistency checks for missing values and errors were made by two authors (K.T. and A.M.). To account for missing data, complete case analysis (CCA) was performed. The missingness mechanism was assessed using Little’s test for Missing Completely at Random (MCAR). The proportion of missing data for each variable was also evaluated. The Shapiro-Wilk test was performed to assess the normality of continuous variables combined with graphical methods. Medians with interquartile ranges (IQR) were used for asymmetrical distributions and simple tabulations for categorical characteristics. Wilcoxon rank-sum test and Pearson’s chi-squared test (or Fisher’s exact test) were used to assess associations between demographic and clinical characteristics and the number of metabolic syndrome components.

Overall survival (OS) was defined as the time from initial evaluation to death from any cause or last contact for alive patients. Progression-free survival (PFS) was defined as the time from initial evaluation to disease progression or death (whichever occurred first) for relapsing patients, or last contact for non-relapsing patients. Survival curves were estimated using the Kaplan–Meier method. Log-rank tests (standard or stratified) were used to test the equality of survivor functions across groups. Cox proportional hazard regression was used for univariate and multivariate analysis to obtain hazard ratios (HR) and their 95% confidence intervals (95% CIs). OS and PFS analyses adjusted for disease stage were also performed. For multivariate analyses, the backward selection procedure with removal criterion (p > 0.10) based on the likelihood ratio test was performed to identify significant variables. Effect modification was assessed by including all pairwise combinations of interaction; assessment for collinearity was also performed. For variables highly correlated, less important ones based on clinical criteria were eliminated from multivariate analysis: albumin and calcium were excluded because of higher proportions of missing data; neutrophil count was excluded because it is incorporated in the neutrophil-to-lymphocyte ratio (NLR); and alkaline phosphatase (ALP) was excluded because it correlated strongly with sodium and potassium. Cancer type was also excluded from PFS multivariate analyses, as the low number of events resulted in unstable estimates.

All statistical tests were two-sided and were performed at a 0.05 significance level. All statistical analyses were performed using the statistical package STATA (StataCorp. 2023. Stata Statistical Software: Release 18. College Station, TX, USA: StataCorp LLC).

## 3 Results

### 3.1 Clinicopathological characteristics

A total of 112 patients were included in the analysis. The majority were male (82.1%), with a median age at diagnosis of 71.9 years (IQR 65.8–79.1). Most patients (92%) had a diagnosis of UBUC, and previous treatment included immunotherapy in 72.3% of patients, chemotherapy in 68.8%, radiotherapy in 45.5%, and surgery in 41.1%. Baseline demographic, clinical, and laboratory parameters of the cohort, stratified by MetS status, are presented in **Table 1** and **Supplementary Table 1**. Seventy patients had information on all variables included in the database and were included in the multivariate analyses. Overall, 42 patients had missing data in at least one covariate considered for multivariable modeling. The proportion of missing data ranged from 0.9% to 22.3% across variables (**Supplementary Table 2**). Little’s MCAR test showed no evidence of systematic missingness (χ² = 109.74, df = 146, p = 0.989). Comparison of baseline characteristics between patients included and excluded from the multivariable analyses showed no significant differences (**Supplementary Table 3**).

**Table 1:** Baseline demographic and clinical characteristics of the study participants, stratified by MetS status.

Metabolic syndrome criteria					
Characteristic	N	Total	0-2	3+	p-value
Median (IQR)					
Age	112	71.9 (65.8-79)	72.5 (62.5-79.1)	71.5 (66.9-77.3)	0.78
BMI	111	27.2 (24.7-30)	25.5 (23.8-27.5)	29.7 (27-31.6)	**<0.001**
N (%)					
Gender	112				0.71
Male		92 (82.1)	51 (81)	41 (83.7)	
Female		20 (17.9)	12 (19.1)	8 (16.3)	
PS	93				0.17
0		80 (86)	43 (91.5)	37 (80.4)	
1		12 (12.9)	4 (8.5)	8 (17.4)	
2		1 (1.1)	0 (0)	1 (2.2)	
Smoking	107				0.53
No		15 (14)	10 (16.4)	5 (10.9)	
Ex		52 (48.6)	27 (44.3)	25 (54.4)	
Current		40 (37.4)	24 (39.3)	16 (34.8)	
Alcohol use	105	11 (10.5)	6 (10.2)	5 (10.9)	0.91
Diabetes	112	75 (67)	27 (42.9)	48 (98)	**<0.001**
Dyslipidemia	112	58 (51.8)	18 (28.6)	40 (81.6)	**<0.001**
Obesity	112	30 (26.8)	6 (9.5)	24 (49)	**<0.001**
Hypertension	112	68 (60.7)	21 (33.3)	47 (95.9)	**<0.001**
Cancer type	112				>0.99
UBUC		103 (92)	58 (82.1)	45 (91.8)	
UTUC		9 (8)	5 (7.9)	4 (8.2)	
Stage	112				0.93
Ta/T1/T2		85 (75.9)	48 (76.2)	37 (75.5)	
T3/T4		27 (24.1)	15 (23.8)	12 (24.5)	
Stage	111				0.45
I/0		16 (14.4)	10 (15.9)	6 (12.5)	
II/IIIA		69 (62.2)	36 (57.1)	33 (68.9)	
IIIB/IV/IVB		26 (23.4)	17 (27)	9 (18.8)	
Treatment					
Chemotherapy		77 (68.8)	45 (71.4)	32 (65.3)	0.49
Immunotherapy		81 (72.3)	47 (74.6)	34 (69.4)	0.54
Radiation		51 (45.5)	30 (47.6)	21 (42.9)	0.62
Surgery		46 (41.1)	22 (34.9)	24 (49)	0.13

MetS, metabolic syndrome; IQR, interquartile range; BMI, body mass index; PS, performance status; UBUC, urinary bladder urothelial carcinoma; UTUC, upper tract urothelial carcinoma.

The bold values represent statistically significant results.

Median BMI of the cohort was 27.2 kg/m² (IQR 24.7–30.0). Nearly half of patients (48.6%) were former smokers, and alcohol use was reported in 10.5%. PS was favorable in most cases, with 86% of patients having ECOG 0.

At diagnosis, 76.6% of patients presented with early-stage disease (I–IIIA), while 23.4% had advanced stage (IIIB–IVB). Similarly, most patients (75.9%) had disease confined to the muscle layer (Ta/T1/T2), while 24.1% presented with tumors extending beyond the muscle layer (T3/T4).

MetS was present in 49 patients (43.8%). Patients with full MetS had higher BMI, diabetes, dyslipidemia, obesity, and hypertension rates compared to those with 0–2 components. No significant differences were observed in age, sex distribution, tumor stage, or treatment modalities between the two groups (**Table 1**).

### 3.2 Overall survival

Survival outcomes were strongly influenced by tumor stage and PS. Patients with extensive disease (IIIB–IVB) had significantly worse OS and PFS compared with those with early-stage disease (0/I) (HR: 7.35, 95% CI 1.72-31.44; p=0.007). Similarly, higher tumor stage (T3/T4 vs. Ta/T1/T2) was associated with poorer outcome (HR 2.18, 95% CI 1.20–3.97; p=0.01). Patients with ECOG PS 1–2 had significantly shorter OS (HR 2.99, 95% CI 1.41–6.32; p=0.004) compared with ECOG 0. Sex was not associated with OS (p=0.61) (**Supplementary Table 4**).

**Figure 1:**
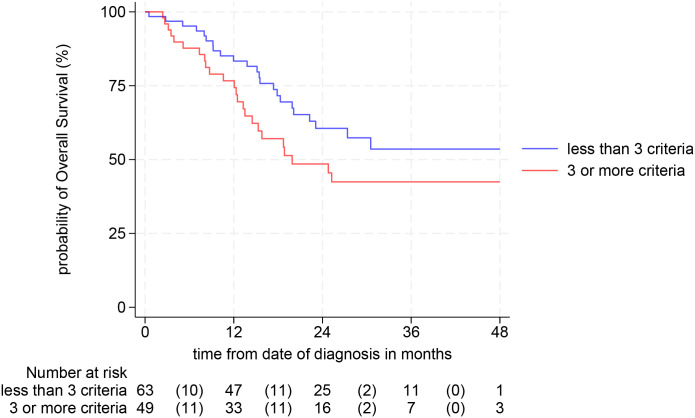
Kaplan–Meier curves for overall survival stratified by metabolic syndrome status, comparing patients with three or more diagnostic criteria versus those with fewer than three criteria.

In univariate analysis, the number of MetS components, diabetes, and BMI were associated with shorter OS (**Supplementary Table 2**). After adjustment for disease stage, MetS significantly predicted worse OS (HR 2.04, 95% CI 1.13–3.68; p=0.018) (**Table 2**; **Figure 1**). The presence of only one component of MetS, irrespective of which criterion was met, was significantly associated with a lower 2-year survival rate (48.5% for one MetS component vs. 60.6% for no MetS components, HR: 5.53, 95% CI 1.26-24.19; p=0.023). In the multivariate model, MetS retained its association with shorter OS (HR 2.27, 95% CI 1.05–4.94, p=0.038) (**Table 3**).

**Table 2:** OS/PFS univariate analyses adjusted for disease stage.

OS analysis adjusted for stage						
Variable		HR	p-value	95% CI		Overall p
MetS criteria	3+ vs 0-2	2.04	**0.018**	1.13	3.68	
Obesity	yes vs no	1.04	0.908	0.52	2.1	
Hypertension	yes vs no	1.96	**0.039**	1.04	3.73	
Dyslipidemia	yes vs no	0.82	0.526	0.45	1.51	
Diabetes	yes vs no	3.34	**0.001**	1.59	6.98	
BMI	25–30 vs <25	0.53	0.07	0.27	1.05	0.19
	>= 30 vs <25	0.66	0.32	0.29	1.51	

PFS analysis adjusted for stage						
Variable		HR	p-value	95% CI		Overall p
MetS criteria	3+ vs 0-2	2.13	**0.024**	1.11	4.1	
Obesity	yes vs no	1.2	0.639	0.56	2.59	
Hypertension	yes vs no	1.54	0.196	0.8	2.98	
Dyslipidemia	yes vs no	1.1	0.777	0.58	2.08	
Diabetes	yes vs no	3.35	**0.002**	1.55	7.22	
BMI	25–30 vs <25	0.53	0.06	0.25	1.03	0.17
	>= 30 vs <25	0.66	0.372	0.27	1.62	

OS, overall survival; PFS, progression-free survival; HR, hazard ratio; 95% CI, 95% confidence intervals; MetS, metabolic syndrome; BMI, body mass index.

The bold values represent statistically significant results.

**Table 3:** OS multivariate analysis.

Variable	HR (95% CI)	*p*-value
MetS criteria		0.038
0-2 (ref)	1	
3+	2.28 (1.05-4.94)	
Stage		0.015
I/0 (ref)	1	0.48
II/IIIA	2.09 (0.27-16.3)	0.078
IIIB/IV/IVB	6.79 (0.8-57.3)	
Hemoglobin	0.81 (0.66-0.99)	**0.04**

OS, overall survival; HR, hazard ratio; 95% CI, 95% confidence intervals; MetS, metabolic syndrome; ref, reference category.

The bold values represent statistically significant results.

**Figure 2:**
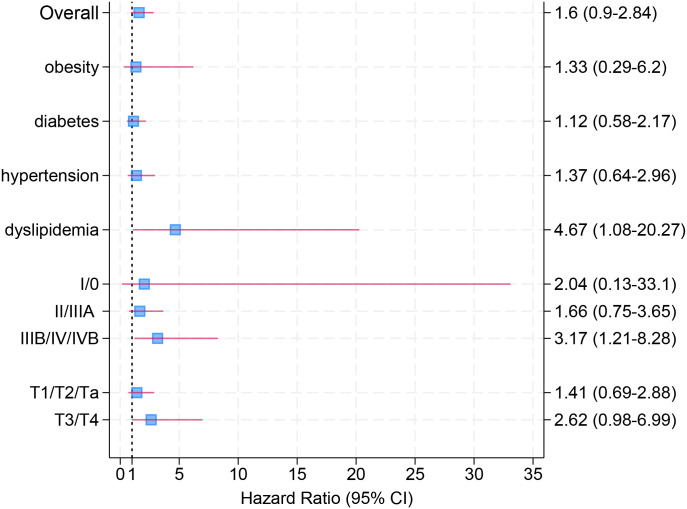
Forest plot of hazard ratios (HRs) for overall survival, showing the overall HR and subgroup analyses by metabolic syndrome components (obesity, diabetes, hypertension, dyslipidemia) and by disease stage categories (I/0, II/IIA, IIB/IV/IVB and T1T2/Ta, T3/T4).

Among the individual MetS components, diabetes had the strongest effect on OS, associated with more than a two-fold increase in mortality risk (HR 2.51, 95% CI 1.21–5.20; p=0.013), and an even stronger effect after adjusting for stage (HR 3.33, 95% CI 1.59–6.98; p=0.001). Hypertension also showed an adverse impact in the adjusted analysis (HR 1.96, 95% CI 1.04–3.73; p=0.039). In contrast, obesity and dyslipidemia did not significantly affect OS (**Tables 2**, **3**; **Figure 2**).

In analyses stratified by treatment modality, MetS was not significantly associated with OS within the different subgroups, and no significant interactions between MetS and treatment modality were observed (all p>0.05).

### 3.3 Progression-free survival

Patients with extensive disease (IIIB–IVB) had significantly worse PFS compared with those with early-stage disease (0/I) (HR: 9.95, 95% CI 2.3-43; p=0.002). Higher tumor stage (T3/T4 vs. Ta/T1/T2) was also associated with poorer PFS (HR 2.10, 95% CI 1.10–4.00, p=0.024). Patients with ECOG 1–2 had significantly shorter PFS (HR 2.33, 95% CI 1.02–5.32; p=0.046) compared with ECOG 0 (**Table 2**).

**Figure 3:**
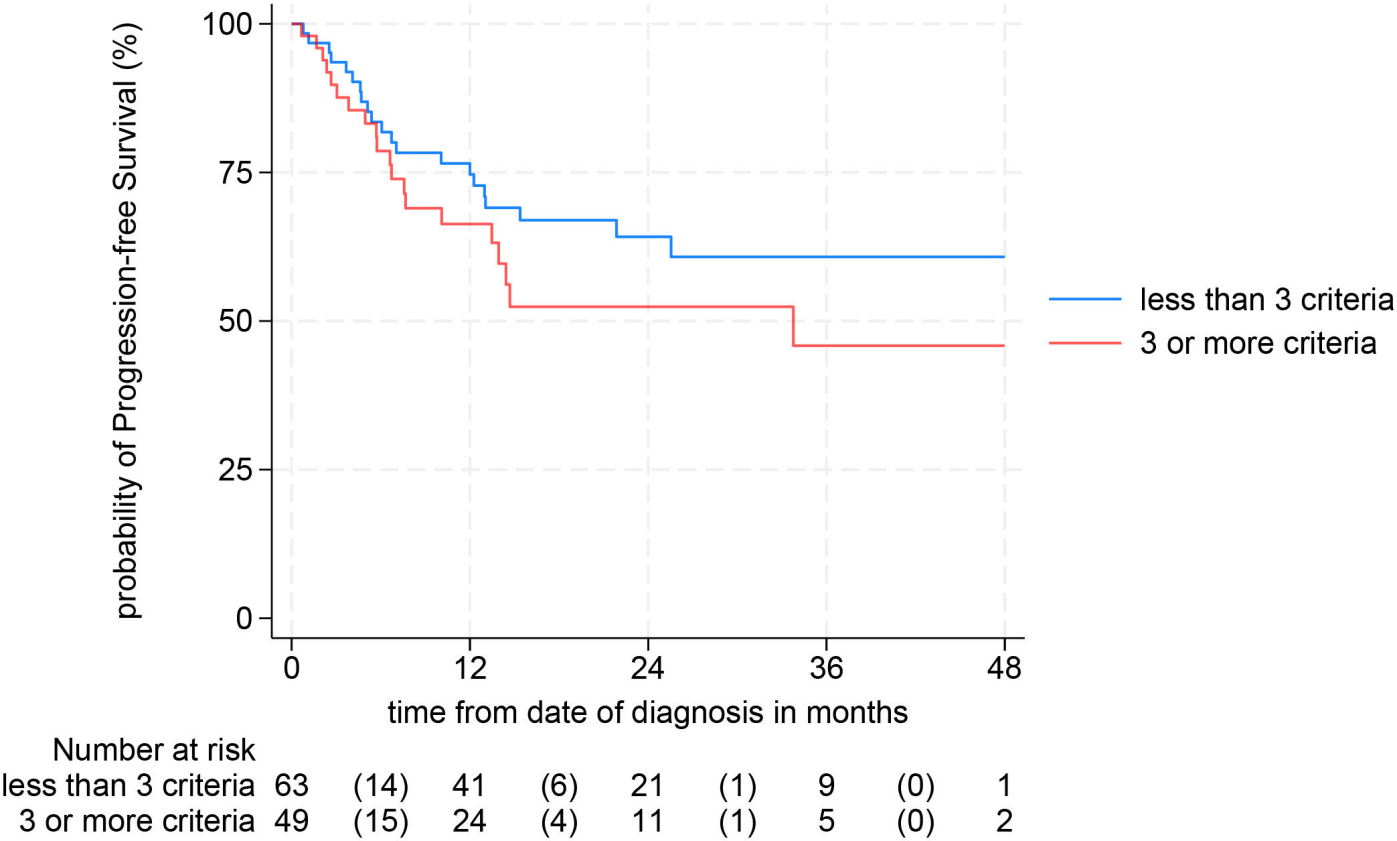
Kaplan–Meier curves for progression-free survival stratified by metabolic syndrome status, comparing patients with three or more diagnostic criteria versus those with fewer than three criteria.

After adjustment for stage, full MetS was significantly associated with poorer PFS compared to that of patients with 0–2 criteria present (HR: 2.13, 95% CI 1.11–4.10, p=0.024) (**Table 2**; **Figure 3**). However, MetS was not retained in the final multivariable model following backward selection using the likelihood ratio test (**Supplementary Table 5**). When MetS was forced into the multivariable model, it remained non-significant (HR 1.59, 95% CI 0.66–3.85, p=0.30; **Supplementary Table 6**).

Among the individual components, diabetes was associated with significantly shorter PFS (HR 2.15, 95% CI 1.02–4.52; p=0.043) and remained significant after adjustment for stage (HR 3.35, 95% CI 1.55–7.22; p=0.002). Neither hypertension, obesity, nor dyslipidemia demonstrated a significant effect on PFS (**Table 2**).

In secondary survival analyses, inflammatory indices were evaluated as exploratory prognostic markers. Higher neutrophil-to-lymphocyte ratio (NLR) and systemic immune-inflammation index (SII) were associated with poorer OS, while SII was also associated with shorter PFS. These associations remained significant after adjustment for disease stage. Detailed results are presented in **Supplementary Table 7**.

## 4 Discussion

This study demonstrates that MetS and its components are predictors of adverse outcomes in UC patients. In our cohort, patients with ≥3 MetS components experienced significantly shorter OS and PFS compared with those with 0–2 components, independent of tumor stage. Diabetes was the strongest and most consistent prognostic factor, while hypertension also predicted poorer OS after adjustment for stage. No differential effects of MetS were observed according to sex or treatment modality, suggesting that its prognostic impact was broadly consistent across patient subgroups.

Previous studies have linked MetS to UC risk, with a two-fold higher risk of UBUC reported in individuals with MetS (7). Similarly, a meta-analysis including over 200,000 participants confirmed a positive association between MetS and UC incidence (14). An association between the number of MetS components and the overall risk of UC was also reported (15). Our findings extend this evidence by demonstrating that MetS independently predicted worse survival in established UC, underscoring its clinical relevance beyond tumorigenesis.

MetS has also been linked to higher tumor grade, advanced stage, and decreased cancer-specific survival (16). Additionally, a Japanese cohort reported an association between MetS and higher T stage, lymphovascular invasion, and high-grade UC (6). Similar findings were described in retrospective series (17). Our study did not show similar associations, possibly due to the relatively low number of patients in each stage. It is important, however, that the adverse impact of MetS on OS and PFS was consistent across all stages examined.

Diabetes emerged as the dominant adverse prognostic factor in our cohort, consistent with previous research linking poor glycemic control to higher recurrence rates and worse prognosis (8, 18). Several studies have also demonstrated higher mortality among people with diabetes undergoing radical surgery (19), while a large meta-analysis of more than 11,000 UTUC patients further confirmed a higher risk of recurrence following nephroureterectomy in this population (20). Antidiabetic therapy might also influence prognosis, as metformin use was associated with improved oncological outcomes (21).

Hypertension also independently predicted poorer OS in our cohort. This aligns with prior studies linking elevated blood pressure (BP) to increased UC incidence and adverse outcomes, independently of tumor characteristics (22, 23). Meta-analytic evidence has further confirmed a dose-dependent relationship between BP and muscle-invasive UC risk (24). A potential additive interaction between systolic BP and a genetic risk score comprising 18 UBUC genetic variants has been reported in men (25).

In contrast, obesity and dyslipidemia were not significantly associated with survival outcomes in our study, aligning with previous inconsistent associations (26, 27). The absence of waist circumference data, requiring the use of BMI as a surrogate measure of obesity, may have contributed to these findings. While this approach has been adopted in other retrospective studies when waist circumference measurements were unavailable (11–13), BMI may not fully capture central adiposity and the metabolic burden associated with visceral fat. Consequently, the contribution of obesity to UC outcomes may have been underestimated in our cohort. Similarly, although dyslipidemia has been implicated in cancer biology through oxidative stress and altered lipid signaling (28), its role in UC progression remains uncertain. Further studies incorporating detailed lipid profiling and direct measures of visceral adiposity could clarify these relationships.

The pathophysiological links between MetS and UC are likely multifactorial. MetS can contribute to a state of persistent, low-grade inflammation and oxidative stress, which could lead to tumor growth and proliferation (29). Additionally, the hyperinsulinemia and insulin resistance often present in MetS can activate insulin-like growth factor signaling pathways, further driving cellular proliferation and cancer progression (30). Although inflammatory indices such as the SII and NLR have previously been associated with MetS (31, 32), in our cohort, they were related to cancer-related outcomes but not MetS status.

Emerging evidence suggests that the interplay between UC and MetS may also be driven by shared genetic and molecular signatures. Key genes such as TAF10 and ABCF2, associated with both UBUC and MetS, appear to play a role in regulating immune responses, and could also potentially serve as clinically relevant biomarkers and therapeutic targets for patients with concurrent UBUC and MetS (33).

The strengths of this study include a well-defined cohort from a large tertiary referral center, detailed assessment of MetS and its components based on consistent clinical definitions, and survival analyses adjusted for tumor stage. These factors enhance the robustness of our findings and add novel data from a European population, which has been underrepresented in the literature. Limitations include the retrospective design, single-center setting, and modest sample size, which may restrict generalizability. In addition, only 70 of the 112 patients were included in the multivariable analyses because of missing data. While complete-case analysis was considered appropriate, the reduced sample size may have limited statistical power and affected the precision of some estimates. Furthermore, some subgroup analyses yielded wide confidence intervals because of the small number of patients and outcome events within certain categories; these estimates should therefore be interpreted cautiously. The lack of waist circumference data, requiring the use of BMI as a surrogate for obesity, is an additional limitation. Future research should aim to validate these findings in larger, multicenter cohorts and explore the underlying biological mechanisms linking MetS to cancer progression.

## 5 Conclusions

Our study suggests that MetS and its components, particularly diabetes and hypertension, are associated with adverse survival outcomes in patients with UC. These findings support the potential role of metabolic health as a prognostic factor in UC and justify further prospective studies to determine whether targeted metabolic interventions may improve patient outcomes.

## Data Availability

The original contributions presented in the study are included in the article/**Supplementary Material**. Further inquiries can be directed to the corresponding author.
