# Supplementary material for: The impact of metabolic syndrome on survival outcomes in urothelial carcinoma: a retrospective cohort study
Source: Front Oncol. 2026 Jun 30;16:1858817. doi: 10.3389/fonc.2026.1858817 (PMC13364600; doi:10.3389/fonc.2026.1858817)
Supplement: Supplementary file 1 [file Table1.docx]

Sup Table S1. Laboratory values of the study cohort stratified by MetS status.

|  |  |  | Metabolic Syndrome criteria | |  |
| --- | --- | --- | --- | --- | --- |
|  |  |  |  | |  |
| Characteristic | N | Total | 0-2 | 3+ | *p*-value |
|  |  |  | Median (IQR) |  |  |
| Hb | 108 | 13.4 (11.9-14.6) | 13.5 (12.2-14.7) | 13 (10.9-14.2) | 0.24 |
| ALP | 101 | 77 (62-95) | 79.5 (64-94.5) | 72 (59-95) | 0.4 |
| WBC | 108 | 7.4 (6.3-9.5) | 7.2 (6.1-9) | 7.6 (6.6-9.9) | 0.098 |
| Neutrophils | 102 | 4.5 (3.8-6) | 4.3 (3.7-5.6) | 4.6 (3.9-6.3) | 0.19 |
| Lymphocytes | 102 | 1.9 (1.5-2.4) | 1.8 (1.5-2.3) | 2.1 (1.6-2.5) | 0.37 |
| PLT | 108 | 248 (205-306) | 242 (198.5-300) | 278 (214-312) | 0.24 |
| Urea | 103 | 37 (29.8-49.2) | 36.9 (31-44) | 39 (28-50) | 0.84 |
| Creatinine | 107 | 0.9 (0.8-1.3) | 0.9 (0.8-1.2) | 1.1 (0.9-1.3) | 0.03 |
| AST | 106 | 15 (13-21) | 16.5 (14-21.5) | 15 (12-19) | 0.064 |
| ALT | 106 | 15.5 (11-22) | 15 (11-19.5) | 16 (11-23) | 0.49 |
| LDH | 73 | 171 (147-211) | 192.5 (157-225.5) | 155 (137-184) | 0.011 |
| Uric acid | 63 | 5.6 (4.6-6.8) | 5.5 (4.3-6.5) | 5.9 (5-7.2) | 0.14 |
| Albumin | 87 | 4.3 (4-4.5) | 4.2 (3.9-4.5) | 4.4 (4-4.5) | 0.89 |
| Total proteins | 74 | 7.1 (6.6-7.4) | 7.1 (6.6-7.4) | 7 (6.6-7.4) | 0.58 |
| Na | 103 | 140 (138-142) | 140 (138-142) | 140 (138-141) | 0.45 |
| K | 103 | 4.5 (4.2-4.8) | 4.4 (4.2-4.8) | 4.6 (4.3-4.8) | 0.23 |
| Ca | 99 | 9.4 (9.2-9.8) | 9.5 (9.2-9.9) | 9.3 (9.2-9.7) | 0.4 |
| P | 68 | 3.1 (2.7-3.7) | 3.1 (2.6-3.4) | 3.2 (2.8-3.8) | 0.35 |
| Mg | 74 | 2 (1.8-2.1) | 2 (1.8-2.1) | 2 (1.8-2.1) | 0.95 |
| TSH | 94 | 1.8 (1.1-2.6) | 1.6 (1.2-2.5) | 1.8 (1-2.6) | 0.96 |
| Fasting glucose | 95 | 104 (95-119) | 100.5 (91-109.5) | 114 (103-145) | <0.001 |
|  |  |  | N (%) |  |  |
| TSH  ≤2.5  >2.5 | 94 | 70 (74.5)  24 (24.5) | 41 (75.9)  13 (24.1) | 29 (72.5)  11 (27.5) | 0.71 |

Hb, hemoglobin; ALP, alkaline phosphatase; WBC, white blood cells; PLT, platelets; AST, aspartate aminotransferase; ALT, alanine aminotransferase; LDH, lactate dehydrogenase; Na, sodium; K, potassium; Ca, calcium; P, phosphorus; Mg, magnesium; TSH, thyroid-stimulating hormone.

Sup Table S2. Extent of missing data across variables included in the analysis.

| **Variable** | **N (non-missing)** | **Missing** | **Proportion of missing (%)** |
| --- | --- | --- | --- |
| Albumin | 87 | 25 | 22.3 |
| PS | 93 | 19 | 17 |
| Ca | 99 | 13 | 11.6 |
| NLR | 102 | 10 | 8.9 |
| K | 103 | 9 | 8 |
| Na | 103 | 9 | 8 |
| Urea | 103 | 9 | 8 |
| Smoking | 107 | 5 | 4.5 |
| PLT | 108 | 4 | 3.6 |
| WBC | 108 | 4 | 3.6 |
| Hemoglobin | 108 | 4 | 3.6 |
| Stage | 111 | 1 | 0.9 |
| BMI | 111 | 1 | 0.9 |
| MetS criteria | 112 | 0 | 0 |

PS, performance status; Ca, calcium; NLR, neutrophil-to-lymphocyte ratio; K, potassium; Na, sodium; PLT, platelets; WBC, white blood cells; BMI, body mass index; MetS, metabolic syndrome.

Sup Table S3. Characteristics of patients with complete data included in multivariate analysis

|  |  |  | **Included** | **Excluded** | |  |
| --- | --- | --- | --- | --- | --- | --- |
|  |  |  | **N=70** | **N=42** | |  |
| **Characteristic** | **N** | **Total** |  | |  | **p-value** |
|  |  |  | Median (IQR) | |  |  |
| Age | 112 | 71.9 (65.8-79) | 71 (64-77) | | 73 (67-79) | 0.26 |
| BMI | 111 | 27.2 (24.7-30) | 27.3 (25-30.7) | | 26.9 (24.2-29.3) | 0.22 |
| NLR | 102 | 2.5 (1.7-3.7) | 2.5 (1.7-3.7) | | 2.5 (1.6-3.6) | 0.65 |
| K | 103 | 4.5 (4.2-4.8) | 4.5 (4.2-4.8) | | 4.4 (4.2-4.7) | 0.66 |
| Na | 103 | 140 (138-142) | 140 (138.4-142) | | 139 (138-142) | 0.49 |
| Ur | 103 | 37 (29.8) | 36 (29-45) | | 41 (30-53) | 0.26 |
| PLT | 108 | 248 (205-306) | 248 (201-304) | | 251 (212-311) | 0.82 |
| WBC | 108 | 7.4 (6.3-9.5) | 7.4 (6.3-8.9) | | 7.3 (6.2-9.8) | 0.85 |
| Haemoglobin | 108 | 13.4 (11.9-14.6) | 13.5 (12-14.6) | | 13.1 (11-14.4) | 0.4 |
|  |  |  |  | |  |  |
|  |  |  |  | |  |  |
| Gender  Male  Female | 112 | 92 (82.1)  20 (17.9) | 59 (84.3)  11 (15.7) | | 33 (78.6)  9 (21.4) | 0.45 |
| PS  0  1  2 | 93 | 80 (86)  12 (12.9)  1 (1.1) | 59 (84.3)  10 (14.3)  1 (1.4) | | 21 (91.3)  2 (8.7)  0 (0) | 0.79 |
| Smoking  No  Ex  Current | 107 | 15 (14)  52 (48.6)  40 (37.4) | 10 (14.3)  33 (47.1)  27 (38.6) | | 5 (13.5)  19 (51.4)  13 (35.1) | 0.92 |
| Alcohol use | 105 | 11 (10.5) | 8 (11.8) | | 3 (8.1) | 0.74 |
| Diabetes | 112 | 75 (67) | 51 (72.9) | | 24 (57.1) | 0.087 |
| Dyslipidemia | 112 | 58 (51.8) | 41 (58.6) | | 17 (40.5) | 0.064 |
| Obesity | 112 | 30 (26.8) | 23 (32.9) | | 7 (16.7) | 0.079 |
| Hypertension (yes) | 112 | 68 (60.7) | 42 (60) | | 26 (61.9) | 0.84 |
| Cancer type  UBUC  UTUC | 112 | 103 (92)  9 (8) | 68 (97.1)  2 (2.9) | | 35 (83.3)  7 (16.7) | 0.025 |
| Stage  Ta/T1/T2  T3/T4 | 112 | 85 (75.9)  27 (24.1) | 55 (78.6)  15 (21.4) | | 30 (71.4)  12 (28.6) | 0.39 |
| Stage  I/0  II/IIIA  IIIB/IV/IVB | 111 | 16 (14.4)  69 (62.2)  26 (23.4) | 10 (14.3)  44 (62.9)  16 (22.9) | | 6 (14.6)  25 (61)  10 (24.4) | 0.98 |
| Treatment  Chemotherapy  Immunotherapy  Radiation  Surgery |  | 77 (68.8)  81 (72.3)  51 (45.5)  46 (41.1) | 48 (68.6)  50 (71.4)  30 (42.9)  31 (44.3) | | 29 (69.1)  31 (73.8)  21 (50)  15 (35.7) | 0.96  0.79  0.46  0.37 |
| MetS criteria  0-2  >3 | 112 | 63 (56.3)  49 (43.8) | 33 (47.1)  37 (52.9) | | 30 (71.4)  12 (28.6) | 0.012 |

Sup Table S4. Univariate analyses for OS/PFS.

| **OS univariate analysis** | | | | | | | |
| --- | --- | --- | --- | --- | --- | --- | --- |
| **Variable** |  | **HR** | **p-value** | **95% CI** |  | **overall p** |  |
| Gender | female vs male | 0.81 | 0.611 | 0.36 | 1.81 |  |  |
| PS | 1-2 vs 0 | 2.99 | **0.004** | 1.41 | 6.32 |  |  |
| PS | 1 vs 0 | 2.76 | **0.011** | 1.26 | 6.05 | **0.006** |  |
|  | 2 vs 0 | 10.12 | **0.028** | 1.28 | 79.96 |  |  |
| TNM Stage | II/IIIA vs I/0 | 2.38 | 0.24 | 0.56 | 10.07 | **<0.001** |  |
|  | IIIB/IV/IVB vs I/0 | 7.35 | **0.007** | 1.72 | 31.44 |  |  |
| T Stage | T3/T4 vs Ta/T1/T2 | 2.18 | **0.01** | 1.2 | 3.96 |  |  |
| MetS criteria | 3+ vs 0-2 | 1.6 | 0.106 | 0.9 | 2.84 |  |  |
| No of metS components | 1 vs 0 | 5.53 | **0.023** | 1.26 | 24.19 | **0.005** |  |
|  | 2 vs 0 | 1.72 | 0.509 | 0.35 | 8.5 |  |  |
|  | 3 vs 0 | 5.79 | **0.018** | 1.36 | 24.66 |  |  |
|  | 4 vs 0 | 1.31 | 0.786 | 0.18 | 9.33 |  |  |
| Obesity | yes vs no | 0.84 | 0.605 | 0.42 | 1.64 |  |  |
| Hypertension | yes vs no | 1.75 | 0.08 | 0.94 | 3.27 |  |  |
| Dyslipidemia | yes vs no | 0.61 | 0.097 | 0.34 | 1.09 |  |  |
| Diabetes | yes vs no | 2.51 | **0.013** | 1.21 | 5.20 |  |  |
| Cancer type | UTUC vs UBUC | 2.03 | 0.106 | 0.86 | 4.79 |  |  |
| BMI | 25-30 vs <25 | 0.45 | **0.017** | 0.23 | 0.87 | **0.036** |  |
|  | >= 30 vs <25 | 0.48 | 0.063 | 0.22 | 1.04 |  |  |
| Alcohol | yes vs no | 0.83 | 0.689 | 0.32 | 2.1 |  |  |
| Smoking | ex vs no | 0.35 | **0.018** | 0.15 | 0.84 | **0.013** |  |
|  | current vs no | 0.85 | 0.695 | 0.38 | 1.92 |  |  |
| Surgery | yes vs no | 1.8 | **0.045** | 1.01 | 3.19 |  |  |
| Radiation | yes vs no | 0.98 | 0.943 | 0.55 | 1.74 |  |  |
| Immunotherapy | yes vs no | 0.85 | 0.611 | 0.45 | 1.59 |  |  |
| Chemotherapy | yes vs no | 2.28 | **0.044** | 1.02 | 5.1 |  |  |
| **PFS univariate analysis** | | | | | | | |
| **Variable** |  | **HR** | **p-value** | **95% CI** | | **overall p** |  |
| Gender | female vs male | 1.02 | 0.966 | 0.45 | 2.3 |  |  |
| PS | 1-2 vs 0 | 2.33 | **0.046** | 1.02 | 5.32 |  |  |
| PS | 1 vs 0 | 2.07 | 0.106 | 0.86 | 4.99 | **0.029** |  |
|  | 2 vs 0 | 10.76 | **0.025** | 1.35 | 85.63 |  |  |
| Stage | II/IIIA vs I/0 | 2.24 | 0.276 | 0.52 | 9.58 | **<0.001** |  |
|  | IIIB/IV/IVB vs I/0 | 9.95 | **0.002** | 2.3 | 43.03 |  |  |
| Stage | T3/T4 vs T1/T2/Ta | 2.1 | **0.024** | 1.1 | 4.01 |  |  |
| MetS criteria | 3+ vs 0-2 | 1.45 | 0.24 | 0.78 | 2.67 |  |  |
| Number of MetS components | 1 vs 0 | 4.84 | **0.039** | 1.08 | 21.68 | **0.032** |  |
|  | 2 vs 0 | 2.14 | 0.344 | 0.44 | 10.31 |  |  |
|  | 3 vs 0 | 5.16 | **0.028** | 1.19 | 22.32 |  |  |
|  | 4 vs 0 | 1.27 | 0.813 | 0.18 | 9.018 |  |  |
| Obesity | yes vs no | 0.87 | 0.708 | 0,.3 | 1.78 |  |  |
| Hypertension | yes vs no | 1.28 | 0.446 | 0.68 | 2.42 |  |  |
| Dyslipidemia | yes vs no | 0.75 | 0.358 | 0.4 | 1.39 |  |  |
| Diabetes | yes vs no | 2.15 | **0.043** | 1.02 | 4.52 |  |  |
| Cancer type | UTUC vs UBUC | 2.52 | 0.054 | 0.99 | 6.43 |  |  |
| BMI | 25-30 vs <25 | 0.42 | **0.014** | 0.21 | 0.84 | **0.03** |  |
|  | >= 30 vs <25 | 0.45 | 0.056 | 0.2 | 1.02 |  |  |
| Alcohol | yes vs no | 1.22 | 0.657 | 0.51 | 2.92 |  |  |
| Smoking | ex vs no | 0.29 | **0.01** | 0.11 | 0.75 | **0.002** |  |
|  | current vs no | 1.04 | 0.924 | 0.45 | 2.44 |  |  |
| Surgery | yes vs no | 1.77 | 0.068 | 0.96 | 3.26 |  |  |
| Radiation | yes vs no | 1.36 | 0.324 | 0.74 | 2.53 |  |  |
| Immunotherapy | yes vs no | 2.1 | 0.073 | 0.93 | 4.75 |  |  |
| Chemotherapy | yes vs no | 3.48 | **0.009** | 1.36 | 8.87 |  |  |

OS, overall survival; PFS, progression-free survival; HR, hazard ratio; 95% CI, 95% confidence intervals; PS, performance status; MetS, metabolic syndrome; BMI, body mass index; UTUC, upper tract urothelial cancer; UBUC, urinary bladder urothelial cancer

Sup Table S5. PFS multivariate analysis

|  | HR (95% CI) | *p*-value | LR test |
| --- | --- | --- | --- |
| PS  1-2 vs 0 | 4.62 (1.55-13.76) | 0.006 |  |
| Stage  II/IIIA vs I/0  IIIB/IV/IVB vs I/0 | 0.49 (0.1-2.46)  3.62 (0.75-17.48) | 0.39  0.11 | <0.001 |
| Smoking  Current vs non/ex | 5.81 (2.24-15.03) | <0.001 |  |
| PLT* | 1.01 (1.002-1.014) | 0.006 |  |
| WBC* | 0.71 (0.58-0.87) | 0.001 |  |

PFS, progression-free survival; HR, hazard ratio; 95% CI, 95% confidence intervals; LR test, Likelihood Ratio test; PS, performance status; PLT, platelets, WPC, white blood cells; *, HR per 1‑unit increase

Sup Table S6. Multivariable analysis for PFS with MetS

|  | **HR (95% CI)** | **p-value** | **LR test** |
| --- | --- | --- | --- |
| PS  1-2 vs 0 | 3.73 (1.17-11.89) | 0.026 |  |
| Stage  II/IIIA vs I/0  IIIB/IV/IVB vs I/0 | 0.48 (0.1-2.36)  3.59 (0.75-17.14) | 0.37  0.11 | <0.001 |
| Smoking  Current vs non/ex | 5.31 (2.02-13.99) | 0.001 |  |
| PLT* | 1.01 (1.003-1.015) | 0.004 |  |
| WBC* | 0.7 (0.56-0.87) | 0.001 |  |
| MetS  3+ vs 0-2 | 1.59 (0.66-3.85) | .3 | .3 |

PFS, progression-free survival; HR, hazard ratio; 95% CI, 95% confidence intervals; LR test, Likelihood Ratio test; PS, performance status; PLT, platelets, WPC, white blood cells; MetS, metabolic syndrome; *, HR per 1‑unit increase

Sup Table S7. Inflammatory indices and OS/PFS.

|  |  | **OS univariate analysis** | | | | **OS adjusted for stage** | | | |
| --- | --- | --- | --- | --- | --- | --- | --- | --- | --- |
| Inflammatory index |  | HR | p-value | 95% CI | | HR | p-value | 95% CI | |
| NLR | >1.76 vs ≤1.76 | 2.37 | **0.029** | 1.09 | 5.15 | 1.41 | 0.351 | 0.68 | 2.93 |
| SII | >580 vs ≤580 | 3.3 | **<0.001** | 1.7 | 6.41 | 2.46 | **0.008** | 1.26 | 4.81 |
| NLR | 1 unit increase | 1.12 | **<0.001** | 1.05 | 1.18 |  |  |  |  |
| PLR | 1 unit increase | 1.001 | 0.067 | 0.99 | 1.002 |  |  |  |  |
| SII | 1 unit increase | 1.000 | **0.001** | 1.0001 | 1.0004 |  |  |  |  |
| **PFS univariate analysis** | | | | | | **PFS adjusted for stage** | | | |
| NLR | >1.76 vs ≤1.76 | 1.41 | 0.351 | 0.68 | 2.93 | 1.6 | 0.212 | 0.76 | 3.38 |
| SII | >580 vs ≤580 | 2.46 | **0.008** | 1.26 | 4.81 | 2.11 | **0.032** | 1.07 | 4.18 |

NLR, neutrophil-to-lymphocyte ratio; SII, systemic immune-inflammation index; PLR, platelet-to-lymphocyte ratio; OS, overall survival; PFS, progression-free survival; HR, hazard ratio; 95% CI, 95% confidence intervals
